# Low Crystallinity of Poly(3-Hydroxybutyrate-co-3-Hydroxyvalerate) Bioproduction by Hot Spring Cyanobacterium *Cyanosarcina* sp. AARL T020

**DOI:** 10.3390/plants10030503

**Published:** 2021-03-08

**Authors:** Kittipat Chotchindakun, Wasu Pathom-Aree, Kanchana Dumri, Jetsada Ruangsuriya, Chayakorn Pumas, Jeeraporn Pekkoh

**Affiliations:** 1 Department of Biology, Faculty of Science, Chiang Mai University, Chiang Mai 50200, Thailand; kittipat_ch@cmu.ac.th (K.C.); wasu.p@cmu.ac.th (W.P.-A.); chayakorn.pumas@cmu.ac.th (C.P.); 2 Department of Chemistry, Faculty of Science, Chiang Mai University, Chiang Mai 50200, Thailand; kanchana.d@cmu.ac.th; 3 Department of Biochemistry, Faculty of Medicine, Chiang Mai University, Chiang Mai 50200, Thailand; jetsada.ruang@cmu.ac.th; 4 Functional Food Research Unit, Science and Technology Research Institute, Chiang Mai University, Chiang Mai 50200, Thailand; 5 Environmental Science Research Center, Faculty of Science, Chiang Mai University, Chiang Mai 50200, Thailand

**Keywords:** polyhydroxyalkanoates, PHB, two-stage cultivation, levulinic acid, response surface methodology, biodegradable plastic, thermal properties, crystallinity

## Abstract

The poly(3-hydroxybutyrate-co-3-hydroxyvalerate) (PHBV) derived from cyanobacteria is an environmentally friendly biodegradable polymer. The low yield of PHBV’s production is the main hindrance to its sustainable production, and the manipulation of PHBV production processes could potentially overcome this obstacle. The present research investigated evolutionarily divergent cyanobacteria obtained from local environments of Thailand. Among the strains tested, *Cyanosarcina* sp. AARL T020, a hot spring cyanobacterium, showed a high rate of PHBV accumulation with a fascinating 3-hydroxyvalerate mole fraction. A two-stage cultivation strategy with sole organic carbon supplementation was successful in maximizing cyanobacterial PHBV production. The use of an optimized medium in the first stage of cultivation provided a 4.9-fold increase in biomass production. Subsequently, the addition of levulinic acid in the second stage of cultivation can induce significant biomass and PHBV production. With this strategy, the final biomass production and PHBV productivity were increased by 6.5 and 73.2 fold, respectively. The GC-MS, FTIR, and NMR analyses confirmed that the obtained PHBV consisted of two subunits of 3-hydroxyvaryrate and 3-hydroxybutyrate. Interestingly, the cyanobacterial PHBV contained a very high 3-hydroxyvalerate mole fraction (94%) exhibiting a low degree of crystallinity and expanding in processability window, which could be applied to polymers for desirable advanced applications.

## 1. Introduction

Biodegradable plastics have become an alternative to traditional petroleum plastics. One of the interesting candidates for such plastics are polyhydroxyalkanoates (PHAs), which are a group of polyester plastics synthesized by microorganisms. Due to their fascinating properties, especially their good biocompatibility, PHAs have attracted attention as a new material in biomedical applications [1,2,3]. The different monomeric structures inside each PHA member indicates the variety of the PHA family. The most typical PHA among the 150 family members is homopolymer poly(3-hydroxybutyrate) (PHB), which is made from the 3-hydroxybutyrate (3HB) subunit [4]. However, the weak mechanical properties of PHB due to its high crystallinity, including brittleness and stiffness, are major disadvantages of PHB for advanced applications. Combining the 3-hydroxyvalerate (3HV) subunit into the PHB backbone improves the mechanical properties of the copolymer of poly(3-hydroxybutyrate-co-3-hydroxyvalerate) (PHBV) [5,6,7]. Hence, PHBV has recently become attractive in biomedical and industrial applications.

Different proportions of 3HV in PHBV confer diverse material features, such as thermal properties, the degree of crystallinity, and mechanical strength, which are favorable for various material applications [8,9,10,11]. It was reported that heterotrophic bacteria can produce low and moderate proportion of 3HV at 24–71 mol% [12,13]. However, PHBV biosynthesis from heterotrophic bacteria requires specific cultivations and complicated carbon substrate supplementation, which have high costs. PHBV biosynthesis from cyanobacteria has been focused on due to the need for simple organic supplementation. Within a few decades, PHBV biosynthesis has taken advantage of cyanobacteria, which is considered a “cyanofactory”, for sustainable secondary metabolized substance production. Cyanobacteria readily assimilate carbon dioxide, solar energy, and trace minerals into cell biomass production without any organic supplementation. There are currently at least 137 cyanobacteria species with the potential to be cultured at an industrial scale, such as *Arthrospira platensis* and *Calothrix scytonemicola* TISTR 8095, which have also been reported in PHA accumulation [14,15]. PHAs, including PHBV, serve as energy storage compounds under unfavorable growth conditions, thereby prolonging biomass production. Nevertheless, a low rate of PHBV production and low degree of 3HV contribution have been occasionally reported in certain cyanobacterial species cultivated under typical cultivation conditions comprising single or multiple carbon substrate supplementation [16,17,18,19].

Two-stage cultivation has been proposed to promote PHBV accumulation under high biomass production. The first stage allows cell growth in an enrichment medium to achieve high biomass in a short time and the harvested cells to be transferred into a media, thus inducing PHBV accumulation, as in second stage [20]. It has been widely reported that heterotrophic conditions involving external stimuli, such as limitations in nitrogen or phosphorus and the supplementation of organic carbon, could promote PHBV accumulation in *Nostoc muscorum* [21,22,23]. Supplementation with organic carbon sources in the second stage of cultivation could include the presence of 3HB and 3HV subunits in PHBV production. The even-carbon-number supply from, e.g., glucose, fructose, and acetate could be transformed to the 3HB subunit backbone while the odd-carbon-number supply from, e.g., propionate, valerate, and levulinic acid could be transformed to the 3HV subunit to be incorporated into the 3HB backbone, turning into PHBV. Engineered *Escherichia coli* demonstrated PHBV accumulation of 34.8% through acetate and propionate supplementation [24]. Moreover, in the wild-type bacterium *Cupriavidus necator*, the production of PHBV with HV content of 54.1% was achieved through fructose and propanol supplementation [25].

It is still unknown how to maximize PHBV production with a higher percentage of 3HV in cyanobacterial cultivation. The objectives of this study were to screen for PHBV-producing cyanobacteria isolated from freshwater environments and solar salterns, as well as the hot spring areas of Thailand, to optimize the maximal growth of the screened cyanobacteria with three macronutrients (NaNO_3_, K_2_HPO_4_, and NaHCO_3_) and to activate PHBV production using two-stage cultivation based on various influencing parameters. It was hypothesized that the more biomass that could be produced during the growth phase of the cyanobacteria, the greater the PHBV that could be accumulated in the cells after induction with nutrient limitations or carbon source supplementation. Advances in the physical properties were achieved and potentially indicate relevant applications.

## 2. Results

### 2.1. Screening of Cyanobacteria for PHBV Production

**Figure 1:**
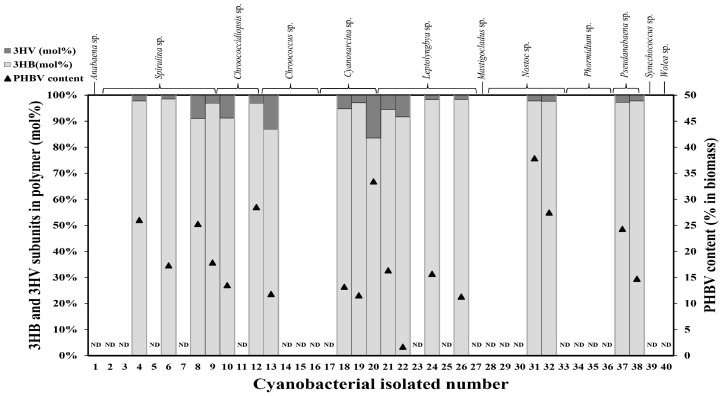
poly(3-hydroxybutyrate-co-3-hydroxyvalerate) (PHBV) production screening in 40 cyanobacterial strains supplemented with 0.4% (*w*/*v*) sodium acetate and 0.4% (*w*/*v*) sodium propionate under heterotrophic conditions. N.D., no detected polymer production by GC analysis.

Among the 40 screened cyanobacterial isolates in 12 genera (Figure 1), there were 23 isolates detectably producing and depositing polyhydroxyalkanoates (PHAs). However, PHBV was found in 18 isolates with content ranging from 0.19% (*Chroococcus* sp. AARL T005) to 37.89% (*Nostoc* sp. AARL UP1). The composition of the 3-hydroxyvalerate (3HV) subunit varied from 0.13 mol% to 4.72 mol% (Table S1). Isolate number 20, *Cyanosarcina* sp. AARL T020, was selected for further experiments (growth profile, two-stage cultivation, and PHBV characterizations) due to having the highest 3HV fraction production (16.42 mol%) and acceptable PHBV contents (33.45%).

**Figure 2:**
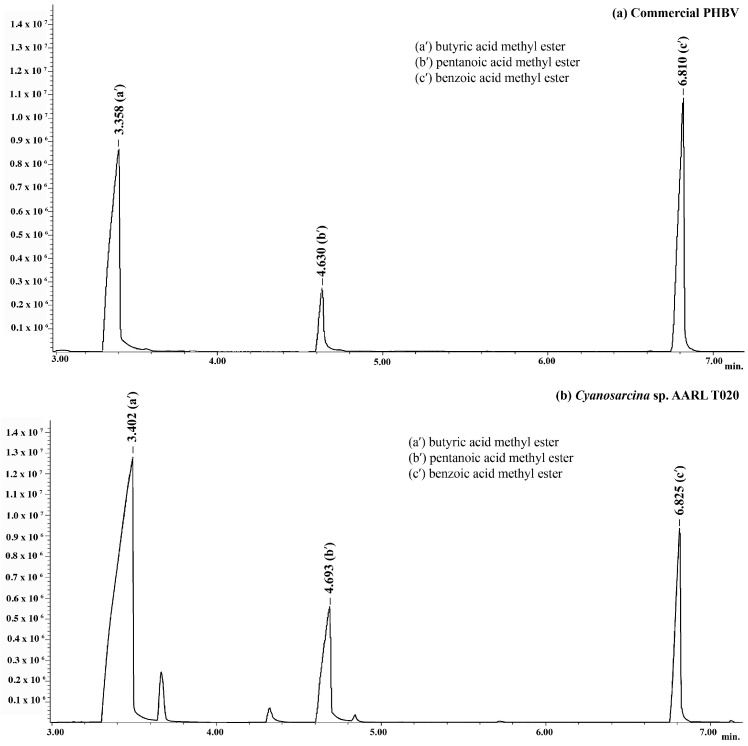
GC spectra of PHBV obtained from (**a**) *Cyanosarcina* sp. AARL T020 compared with (**b**) the commercial standard PHBV.

The gas chromatogram of the PHBV obtained from *Cyanosarcina* sp. AARL T020 after methanolysis show three major peaks along with the peaks of the commercial standard PHBV. The peaks at 3.402 and 4.693 min represent, respectively, the 3HB and 3HV subunits in *Cyanosarcina* sp. AARL T020 (Figure 2). Benzoic acid was used as the internal standard, peaking at 6.825 min.

### 2.2. The Growth of Cyanobacteria

**Figure 3:**
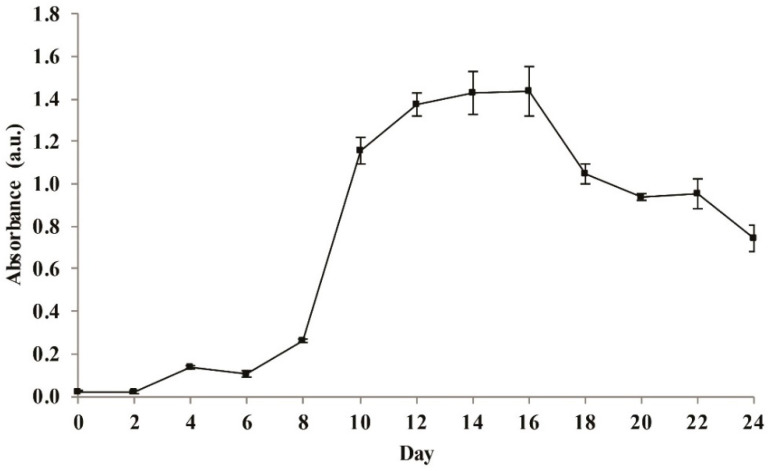
The phototrophic growth curve of *Cyanosarcina* sp. AARL T020 in the BG11 medium.

The cyanobacterial growth reflecting biomass production was obtained from the absorbance at 560 nm at each time point (Table S3). *Cyanosarcina* sp. AARL T020 entered the stationary phase at day 12. The maximum biomass production was observed at day 16, while the death phase was observable by day 18 (Figure 3). The cultivation period for further studies was, therefore, chosen as 14 days.

### 2.3. Model Fitting of First-Stage Cultivation

The CCRD suggested five levels for the three relevant factors (Table 1) based on the actual data, including six axial points, eight cubic points, and three central points. In total, 17 culture experiments were performed to achieve the stationary phase again to determine biomass production (Table S4). The area of dry biomass production values ranged from 0.55 to 0.98 g L^−1^, indicating the optimum point of biomass production. The quadratic polynomial (Equation (1)) represents the interrelation among the three independent variables X1, X2, and X3, which are the NaNO_3_, K_2_HPO_4_, and NaHCO_3_ concentrations, respectively, and the response (Y), which is biomass production.

(1) $$Y=0.9224+0.0572X_{1}+0.0853X_{2}+0.0186X_{3}-0.0037X_{1}X_{2}-0.0137X_{1}X_{3}+0.0362X_{2}X_{3}-0.0350X_{1}^{2}-0.0810X_{2}^{2}-0.0598X_{3}^{2}$$

The biomass production values predicted by the quadratic polynomial Equation (1) were directly compared with the actual biomass production values from the experiment. The results indicated values consistent with the model, with a coefficient of determination (R^2^) of 0.9045.

The analysis of variance (ANOVA) (Table 2) demonstrated the predictability of the equation model with the tested variables. The model showed significant prediction with an F-value of 7.37. Both the linear effect and second-order effect of NaNO_3_ plus K_2_HPO_4_ and K_2_HPO_4_ plus NaHCO_3_ were significant (*p* < 0.05). The lack of fit corresponding to pure errors was not significant.

### 2.4. Effect of Independent Variables and Model Verification

**Figure 4:**
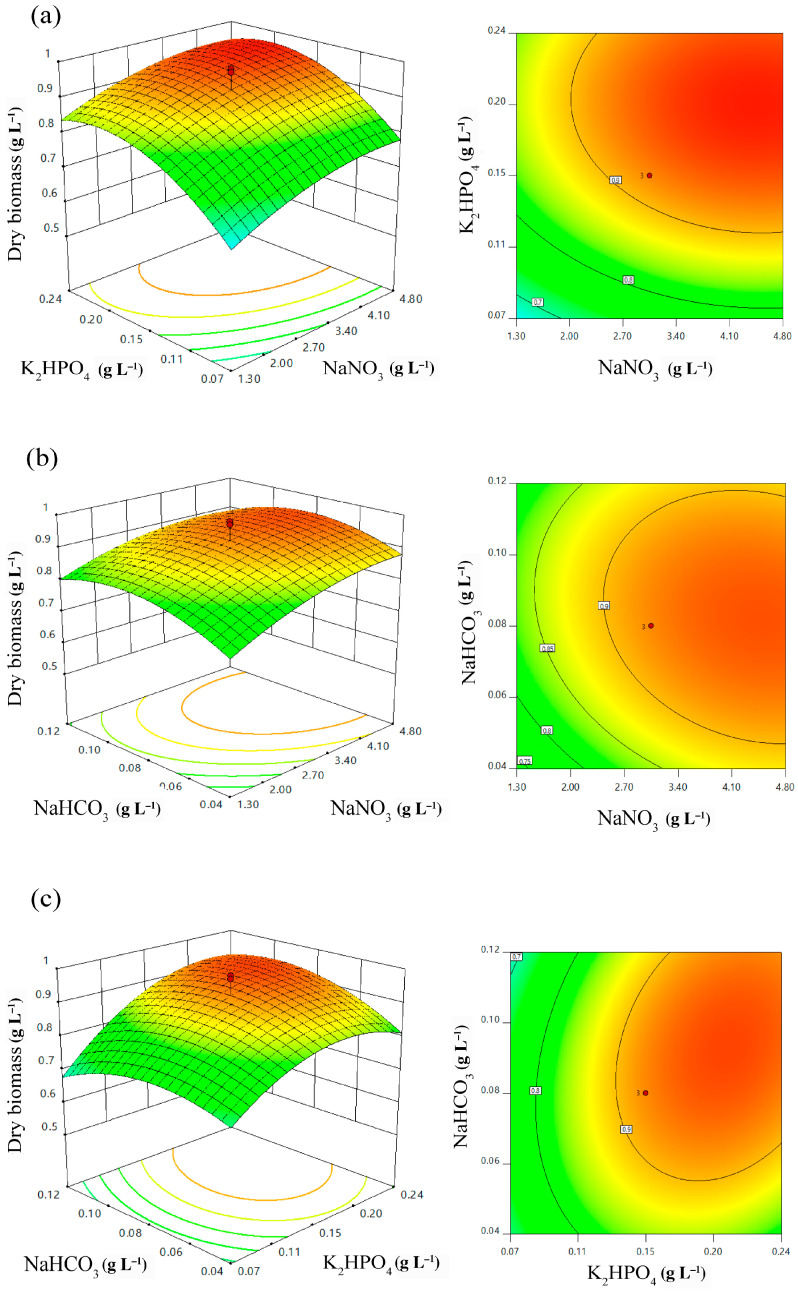
Response surface 3D plots and contour plots of biomass production showing the interaction effects of (**a**) NaNO_3_ and K_2_HPO_4_; (**b**) NaNO_3_ and NaHCO_3_; and (**c**) K_2_HPO_4_ and NaHCO_3_.

The 3D surface and sequence contour plots (Figure 4) were illustrated using the quadratic polynomial model. The interactions between two independent values, with one featuring a fixed value from the other, were plotted against dry biomass production. The interaction between NaNO_3_ and K_2_HPO_4_ with fixed NaHCO_3_ resulted in a significant increase in biomass production, where the concentration of NaNO_3_ increased from 0.10 to 3.05 g L^−1^ (Figure 4a) in accordance with the increase in K_2_HPO_4_ concentration. However, the increase of NaNO_3_ and K_2_HPO_4_ above the zero level indicated a slightly decreasing trend in terms of biomass production. Likewise, the interaction between the NaNO_3_ and NaHCO_3_ concentrations with a fixed concentration of K_2_HPO_4_ showed that the increases in both NaNO_3_ and NaHCO_3_ concentrations slightly promoted biomass production (Figure 4b). Consequently, the optimal NaNO_3_ and NaHCO_3_ concentrations might be the definite constants closest to the point prior to entering the declining state. Similarly, the interaction between the K_2_HPO_4_ and NaHCO_3_ concentrations under a fixed NaNO_3_ concentration showed the K_2_HPO_4_ concentration to range from 0.01 to 0.30 g L^−1^, while the NaHCO_3_ concentration ranged from 0.01 to 0.15 g L^−1^ (Figure 4c). Taken together, according to these experimental plots, the optimal concentrations of NaNO_3_, K_2_HPO_4_, and NaHCO_3_ are 4.35, 0.20, and 0.09 g L^−1^, respectively.

A triplicate experiment was performed with the optimal concentrations of NaNO_3_, K_2_HPO_4,_ and NaHCO_3_ to verify the model. We observed non-significant results between the outcome of the model prediction for biomass production, which was 0.969 g L^−1^, and the verified experimental outcome for biomass production, which was 1.220 ± 0.96 g L^−1^ (Table 3).

### 2.5. Second-Stage Cultivation with PHBV Production Parameters

Second-stage cultivation of *Cyanosarcina* sp. AARL T020 for PHBV production with induction by supplementation with different carbon sources, including glucose, glycerol, sodium acetate, sodium propionate, and levulinic acid at 0.4% (*w*/*v*), was carried out for another 14 days under heterotrophic conditions (Table 4). The highest PHBV content of 69.18% was observed under levulinic acid supplementation with 94.09 mol% of the 3HV fraction. Sodium propionate supplementation also showed 3.28% PHBV content with 79.08 mol% of the 3HV fraction. In contrast, supplementation with sodium acetate, glucose, and glycerol did not induce PHBV production but instead led to PHB accumulation (2.83–17.91%). Nitrogen and phosphorus deprivation offered no benefits for the production of any polymer type. Moreover, a further increase in biomass production was observed under levulinic acid (1641 mg L^−1^), sodium acetate (1515.5 mg L^−1^), and glycerol (1207 mg L^−1^) supplementation. The PHBV productivity under levulinic acid supplementation was calculated from the PHBV content and biomass production. It was found that the highest PHBV productivity was 81.29 mg L^−1^ day^−1^.

### 2.6. Surface Analysis of Extracted PHBV

**Figure 5:**
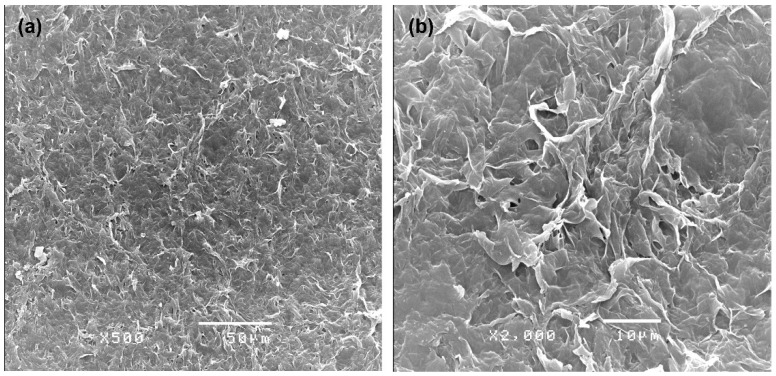
Scanning electron micrographs of the surface of the PHBV film extracted from Cyanosarcina sp. AARL T020 at different magnifications: (**a**) 500× and (**b**) 2000×.

Scanning Electron Microscopy (SEM) was used to analyze the upper surface (e.g., the side exposed to the air) of the extracted PHBV film. Figure 5a presents the smooth base surface of the PHBV polymer with a compact arrangment. However, Figure 5b, at higer magnification, reveals the randomly rugged structures combined with small pore sizes.

### 2.7. Chemical Characterization of Extracted PHBV

**Figure 6:**
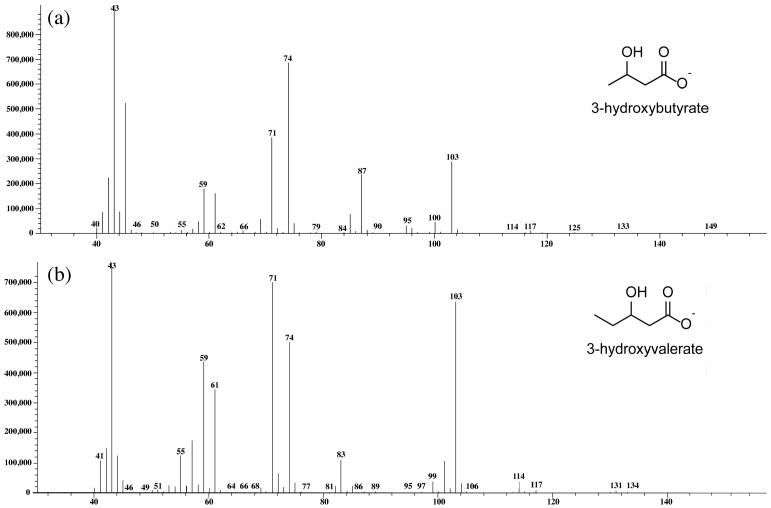
Mass spectrometry analysis of PHBV extracted from *Cyanosarcina* sp. AARL T020. The (**a**) 3-hydroxybutyrate (3HB) subunit and (**b**) 3-hydroxyvaryrate (3HV) subunit were compared with the mass spectra MS library (NIST 11).

Mass spectrometry confirmed that the extracted polymer was PHBV comprising 3-hydroxybytyrare (3HB) and 3-hydroxyvalerate (3HV) (Figure 6). Using the MS library (NIST 11), we were able to confirm the presence of the 3HB (Figure 6a) and 3HV subunits (Figure 6b) in the extracted polymer produced by *Cyanosarcina* sp. AARL T020, indicating PHBV under optimized growth conditions followed by levulinic acid supplementation.

**Figure 7:**
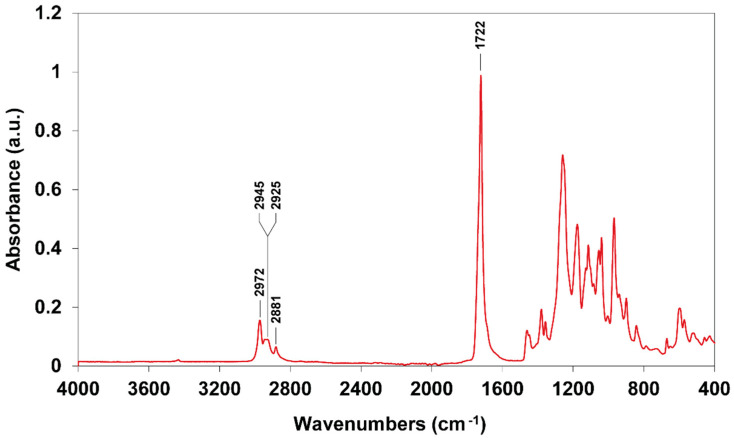
Fourier transform infrared spectroscopy (FTIR) spectra of PHBV extracted from *Cyanosarcina* sp. AARL T020.

The Fourier transform infrared spectroscopy (FTIR) spectra revealed the different functional groups associated with PHBV (Figure 7). The peak at 2972 cm^−1^ is represented by the asymmetric stretching mode of the methyl (-CH_3_) group. The bands in the 2925–2945 cm^−1^ region indicate the antisymmetric stretching mode of methylene (-CH_2_). The peak at 2881 cm^−1^ corresponds to the symmetric stretching mode of CH_3_. The apparent peak at 1722 cm^−1^ was assigned to the C=O stretching group, while the bands in the 800–1500 cm^−1^ region represent the CH_3_ and CH bending vibrations coupled with C-O-C and C-C stretching vibrations.

**Figure 8:**
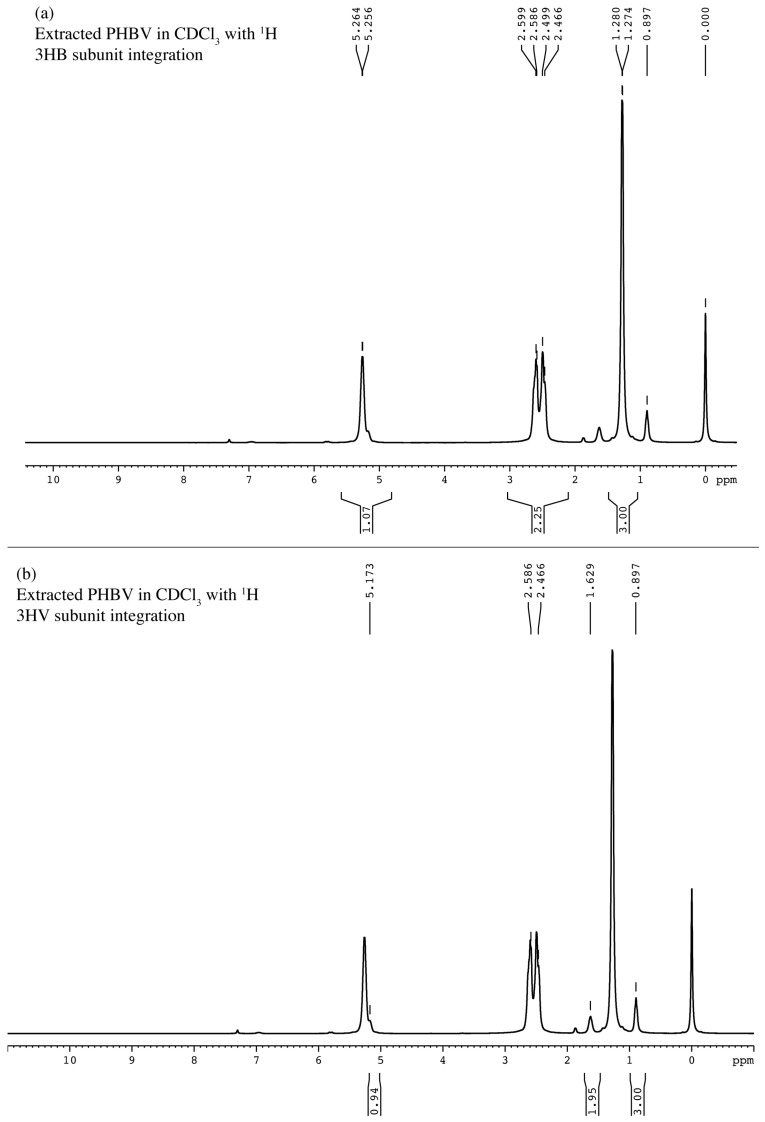
^1^H-NMR spectrum of the PHBV extracted from *Cyanosarcina* sp. AARL T020: (**a**) 3HB subunit and (**b**) 3HV subunit integrations.

The 1H-NMR spectrum of the extracted polymer dissolved in deuterochloroform exhibited seven clearly vibrating proton signals in a range of (δ) 0.897–5.264 ppm, including the 3HB subunit and 3HV subunit (Figure 6). The 3HB subunit (Figure 8a) presented double signals at 5.256 and 5.264 ppm of asymmetric carbon (-CH) belonging to the chiral carbon atom. The multiplet resonance at 2.586–2.599 and 2.466–2.499 ppm indicates diastereotopic methylene (-CH_2_) protons. The doublet signals at 1.274–1.280 ppm indicate methyl protons (-CH_3_). Similarly, the 3HV subunit (Figure 8b) presents chiral carbon (-CH) and methylene proton (-CH_2_) peaks. In addition, the two singlets at 0.897 and 1.629 ppm indicate ethyl protons (-CH_2_-CH_3_).

**Figure 9:**
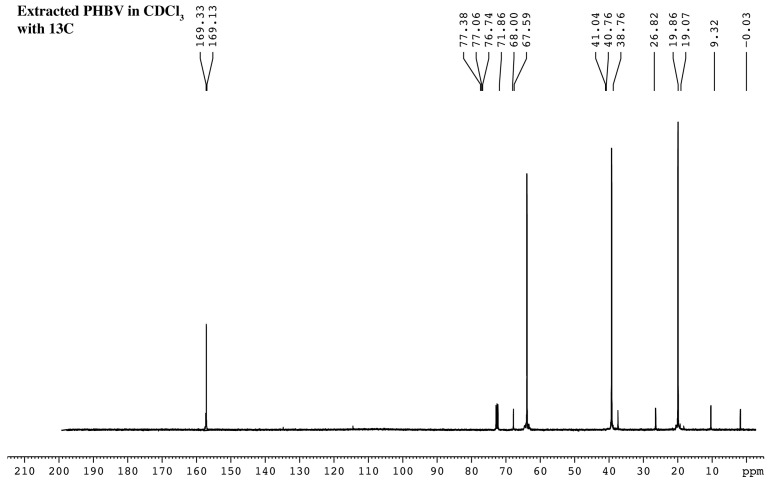
^13^C-NMR spectrum of the PHBV extracted from *Cyanosarcina* sp. AARL T020.

The 13C-NMR spectrum was also observed for vibrating carbon signals ranging from (δ) 9.32 to 169.33 ppm. The doublet signal between 169.13 and 169.33 indicates carboxylic carbon (C=O), while the signal at 67.59–68.00 ppm indicates asymmetric carbon as –CH. The signal around 40.76–41.04 ppm is -CH_2_. The vibrating signals at 19.07–19.86 ppm and 9.32 ppm are methyl carbon (-CH_3_) and ethyl carbons (-CH_2_-CH_3_), respectively (Figure 9). Tetramethylsilane was applied as the internal standard (peak at 0 ppm) in all tests.

Gel permeation chromatography (GPC) was used to measure the size of the extracted PHBV. The weight-average molecular weight (*Mw*) and number-average molecular weight (*Mn*) were determined to be 63.9 kDa and 42.0 kDa, respectively, and a polydispersity index (PDI) of 1.51 was observed.

### 2.8. Thermal Properties of PHBV

**Figure 10:**
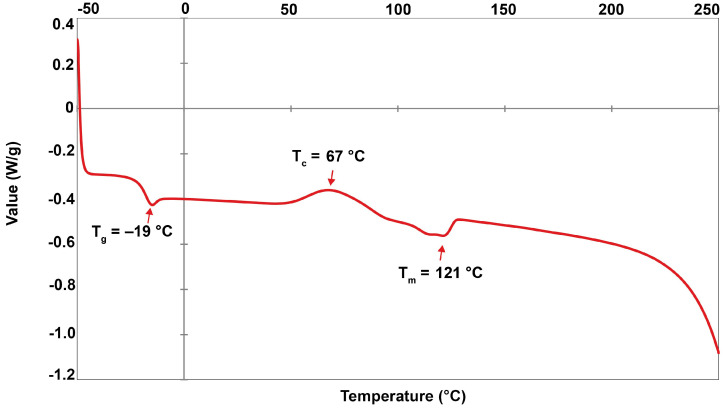
Differential Scanning Calorimetry (DSC) of PHBV from *Cyanosarcina* sp. AARL T020; *Tm*, melting temperature; *T_c_*, thermal crystallization temperature; *T_g_*, glass transition temperature.

Differential scanning calorimetry was used to determine the thermal properties of the extracted PHBV polymer. Figure 10 shows the DSC thermogram in a temperature range from −50 °C to 250 °C. The melting temperature (Tm), thermal crystallization temperature (Tc), and glass transition temperature (Tg) were 121.17, 67.33, and −19.01 °C, respectively. Table 5 outlines the degree of crystallinity (Xc) estimated (equation 2) based on the melting enthalpy of ΔH_m_ (7.07 J g^−1^) and the melting enthalpy of 100% crystallinity for PHB ΔH°_m_ (146 J g^−1^) at 4.84%. In addition, the crystalline fraction (CF) was calculated (Equation 3) based on the crystallization enthalpy of the extracted PHBV ΔH_c_ (6.05 J g^−1^) at 85.57%.

**Figure 11:**
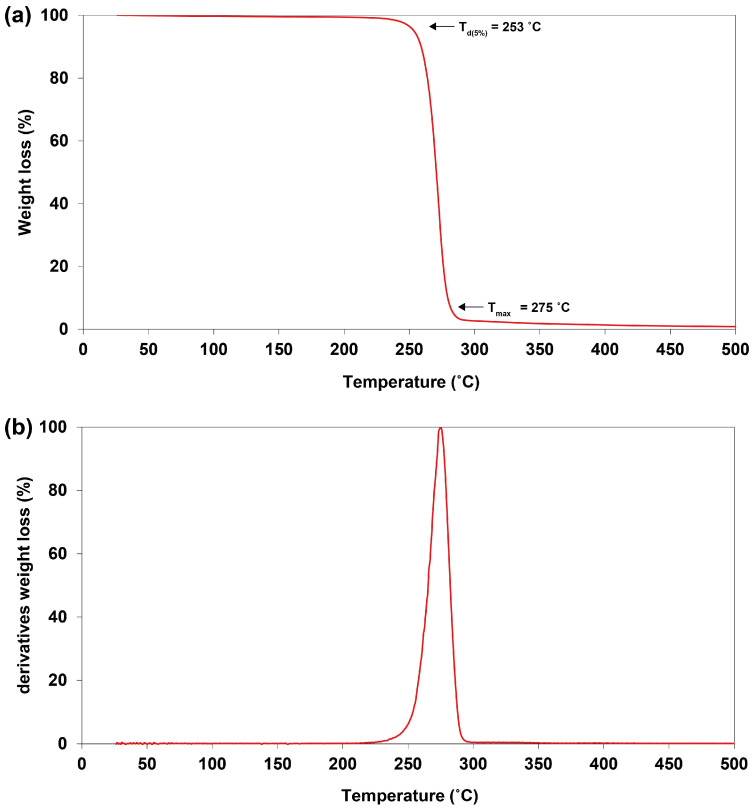
Thermogravimetric analysis (TGA) of PHBV from *Cyanosarcina* sp. AARL T020: (**a**) the weight loss curve; *T_d(5%)_*, the temperature at 5% weight loss; *T_max_*, the maximum degradation temperature; (**b**) derivatives of the weight loss curves.

Thermogravimetric analysis (TGA) was used to determine the thermal stability of the extracted PHBV. A one-step process of degradation was observed. The temperature of 5% weight loss (*T_d_*_(5%)_) began around 253 °C (Figure 11a). The maximal degradation temperature (*T_max_*) was detected at 275 °C, corresponding to the peak of the derivative of the weight loss curve (Figure 11b).

## 3. Discussion

Accumulated PHAs are hypothesized to be one of the intracellular storage substances under unfavorable environmental or stress conditions, such as nutrient limitations. Limitations in macronutrients like nitrogen or phosphorus could promote PHA production in a number of cyanobacteria and bacteria [11,12,18,26]. Furthermore, even-carbon-number (acetate) and odd-carbon-number (propionate) supplementation under heterotrophic condition could induce PHBV production due to the abundance of substrate pools including 3HB-CoA and 3HV-CoA with the aid of PHA synthase [27]. The forty cyanobacterial isolates obtained from freshwater environments, halophilic fields, and hot spring areas showed differential PHA production, including homopolymer–PHB and heteropolymer-PHBV. The highest PHBV production was found in isolate number 31, *Nostoc* sp. AARL UP1, at 37.89%, although the low 3HV fraction (2.21 mol%) still hindered PHBV’s greatest physical properties. Hence, isolate number 20, *Cyanosarcina* sp. AARL T020, was alternatively chosen as a candidate for in-depth study due to having the highest 3HV fraction production (16.42%) and acceptable PHBV production (33.45%). However, some cyanobacterial isolates showed no ability to accumulate PHAs under the induced conditions. The variation in PHA accumulation could be explained by the divergence of cyanobacterial evolution [19].

It is agreed that macronutrients strongly influence the photosynthesis, chemical composition, cellular metabolism, and biomass production in cyanobacteria [28]. The effect of the macronutrient ratio has been reported to have a direct result on biomass production. Our study showed that the ratio of nitrogen-to-phosphorus (N:P) in the trace element BG11 medium was 37.50, while that under optimized conditions was 21.75 (Table S2). The N:P ratio is considered one of the main parameters to determine cyanobacterial growth [29,30]. The optimum N:P ratio of cyanobacteria varies by species from 8.20 to 45.0 [31]. A decline of the N:P ratio tends to favor the development of cyanobacterial biomass [32,33]. Additionally, the amount of inorganic carbon in the environment is crucial to drive the photosynthesis rate in cyanobacteria. A 4.5-fold increase of the carbon source (NaHCO_3_) in an optimized BG11 medium could enhance the bicarbonate (HCO^−3^) pool in the cultural medium, thereby contributing to biomass production via the Calvin–Benson cycle [34]. In addition to the N:P ratio, the carbon ratio of those sources is vital for each cyanobacterium’s growth [35,36]. It is strongly suggested that optimization of the nutrient concentrations in cyanobacterial cultivation is done to accomplish maximal cyanobacterial biomass production. One explanation for the high concentrations of NaNO_3_, K_2_HPO_4_, and NaHCO_3_ reducing biomass production might be cellular toxicity [37,38,39]. Whether macronutrients are beneficial or harmful to biomass production clearly depends on dosage, which is indicated by hormesis. Hormesis is a characteristic of biphasic biological responses, i.e., a low dose demonstrates a positive effect-promoting response, while a high dose exhibits a negative effect-inhibiting response [37].

The strong 4.88-fold increase of 0.250 g L^−1^ up to 1.220 g L^−1^ in biomass production compared to before optimization was manipulated by using the response surface methodology (Table 3), suggesting that biomass optimization under the CCRD model can be used to achieve the maximum efficiency in cyanobacteria biomass production. Our optimization process using the response surface methodology was consistent with the aforementioned findings. Morowvat and Ghasemi reported that the biomass yield of *Dunaliella salina* experienced a 1.75-fold increase after optimizing the nitrogen, phosphorus, and carbon source concentrations [40]. Similarly, Zhai et al. determined that the biomass production of *Spirulina platensis* could increase to 262.50 mg L^−1^ by modifying pH and light intensity and employing daily illumination time optimization [41].

The variations in PHA production under disparate nutrient limitations and organic carbon supplementation are exceedingly fascinating, especially to uncover the environmental responses to cyanobacteria. Our study determined the maximum production of high-HV-fraction PHBV in *Cyanosarcina* sp. AARL T020 under second-stage cultivation (Table 4). The yield of PHBV production containing 94.09 mol% 3HV was 69.18% with a rate of 81.30 mg L^−1^ day^−1^ after 0.4% *w*/*v* levulinic acid activation. This is consistent with the industrial PHBV-producing bacterium, *Cupriavidus necator,* which produces 81.2% PHBV, comprising a 53.9 mol% HV fraction after levulinic acid addition as the co-substrate [42]. Due to the structural analog of levulinic acid as pentanoic acid, it has been assessed as a secondary substrate in PHA biosynthesis pathway. How levulinic acid triggers PHBV accumulation can be explained by the metabolism of the acid. The catabolic pathway of levulinic acid in *Pseudomonas putida* KT2440 begins with the transformation to levulinyl-CoA, which requires at least two ATP and one reducing equivalent to produce 3-hydroxyvaleryl–CoA. The β-oxidation of 3-hydroxyvaleryl–CoA to acetyl-CoA and propionyl-CoA could produce the most crucial substrates that combine into the PHBV polymer [43]. Propionate supplementation could serve as an alternative to promote PHBV accumulation. Our result is in agreement with previous studies in which propionate addition was able to promote PHBV content in *Nostoc muscorum* Agardh due to the abundance of propionyl-CoA inside the cell [16]. Homopolymer–PHB accumulation was also found in the case of even-carbon-number supplementation, including glucose and acetate. This result is consistent with earlier studies in which the presence of acetate under culture conditions was able to stimulate PHB production in cyanobacteria, such as *Arthrospira platensis* UMACC 161 and *Synechocystis* sp. UNIWG, with yields of 10% and 15%, respectively [44]. However, glycerol, an odd-carbon-number substrate, activated homopolymer–PHB accumulation due to the metabolic pathway conversion in PHB biosynthesis [45]. Unlike carbon source supplementation, nitrogen and phosphorus were reported to have an effect on PHA accumulation in cyanobacteria, such as in *Calothrix scytonemicola* TISTR 8095 and *Synechocystis* sp. PCC 6803 [15,46]. These findings contrast with our results, in which *Cyanosarcina* sp. AARL T020 featured no PHA accumulation. Even though the PHA mechanism in cyanobacteria is not completely understood, the most widely accepted hypothesis of the role of PHAs under unfavorable growth conditions is as reducing equivalents [47]. Physiological stresses, such as nitrogen or phosphorus limitations, were found to direct metabolic flux to PHA accumulation under photoautotrophic conditions [16,48,49,50]. In our study, second-stage cultivation was established under heterotrophy. The deficiency of light energy conversion could be related to the amount of energy compounds that provide energy to drive metabolism in photosynthetic organisms. PHA accumulation was thus unable to be detected under these conditions.

The chemical structure identification of PHBV from *Cyanosarcina* sp. AARL T020 was confirmed by mass-spectroscopy, FTIR, and NMR analysis. Our findings correspond to the studies in [16,22,23]. The surface analysis revealed a smooth base material. Even though the addition of the 3HV subunit into the PHB backbone improved the material’s brittleness [16], rugged structures still appeared. This finding could have resulted from the influence of solvent evaporation during the film casting process.

Thermal properties are a crucial consideration when using material in advanced applications. Differential scanning calorimetry (DSC) is regularly used to detect the significant heat flow of polymers and thermal stability. Table 5 indicates indicates the lowest glass transition temperature (*Tg* = −19 °C) and a decrease in the melting temperature (*T_m_* = 121 °C) of the PHBV biosynthesized from *Cyanosarcina* sp. AARL T020 compared to commercial PHBV [51]. This most likely occurred due to the extremely high 3HV fraction (94.09 mol%). *T_g_* and *T_m_* are particularly correlated with polymer chain mobility, changing from a glassy to rubbery state and a rubbery to liquid state, respectively. Both temperature points represent the amount of endothermic energy required to be used in each transition state. Hence, less endothermic energy will be required to move the polymer chains of the biosynthesized PHBV in the amorphous phase [52]. These results agree with previous findings: A greater 3HV fraction leads to a markedly lower *T_g_* and *T_m_* of PHBV [5,6,7]. DSC can also conveniently identify the significant heat flow caused by the exothermic energy of the crystallization process (*T_c_*). *T_c_* is one of the parameters used to determine the crystallization behavior of polymers [52]. A decrease in *T_c_* (67 °C) was also observed in our biosynthesized PHBV compared to the commercial PHBV, with 8 mol% of 3HV [51]. This suggests that a very high 3HV mole fraction could reduce the nucleation of PHBV and trigger crystallization at a lower temperature. Additionally, the exothermic peak of the extracted PHBV was expansive, which suggests that the nucleation and crystal formulation were slower [53]. A lower *T_c_* corresponds to significantly decreased melting enthalpy (Δ*H_m_* = 7.07 j g^−1^), indicating a remarkably lower *Xc* (4.84%). Conversely, a high value of the crystalline fraction (*CF*) (85.57%) was calculated using the inverse ratio with *X_C_*. This finding agrees well with the previous study, suggesting that a lower *X_C_* corresponds to a higher *CF* [54]. This behavior could be related to the molecular weight of the polymer. The polymer with low crystallinity also had a shorter polymeric chain length [55]. In the DSC run, the shortened polymeric chain of the biosynthesized PHBV from *Cyanosarcina* sp. AARL T020 (42.0–63.9 kDa) was readily capable of crystallization, leading to an increase in CF. Therefore, the low crystallinity of biosynthesized PHBV likely corresponds to its shortened polymeric chain and high crystalline fraction.

The reduction in the thermal properties (e.g., *Tg*, *Tm*, *Tc*, ΔH_m,_ and *Xc*) and average molecular weight (e.g., *Mw* and *Mn*) agree with previous reports on PHBV, with the 3HV ranging from 6.50 to 96.20 mol% (Table 5). The enlarged incorporation of 3HV, as a consequence of levulinic acid supplementation, into the PHBV polymer not only decreased the thermal properties of the polymer but also scaled down the range of the melting process (from 121 to 253 °C), which generated lower heating demand during the manufacturing process compared to commercial PHBV and PHB [51,56]. Furthermore, the thermogravimetric analysis (TGA) showed a high-value maximum degradation temperature (284 °C). This finding demonstrates the polymer’s ability to withstand a high temperature, thereby benefiting its thermal stability. A similar observation was reported by Bhati and Mallick [23].

Beyond levulinic acid supplementation leading to high yield PHBV production in *Cyanosarcina* sp. AARL T020, two-stage cultivation was shown to facilitate maximal biomass yield. This strategy demonstrates the noteworthy capabilities of PHBV production, not only in cyanobacteria but also in bacteria, as previously reported [57]. Furthermore, this strategy could be implemented in the production of several secondary metabolized substances, such as astaxanthin in *Haematococcus pluvialis* JNU35, extracellular polymeric substances (EPS) in *Arthrospira* sp., docosahexaenoic acid in *Crypthecodinium cohnii*, and lipid production in *Dunaliella tertiolecta* [58,59,60,61]. From an economic point of view, levulinic acid is a renewable co-product that can be produced cost-effectively at an industrial scale, as the production costs of levulinic acid can be as low as $0.04–$0.10/lb depending on the scale of operation [62,63,64]. Levulinic acid can serve as a cheap alternative to conventional biosynthesis substrates and use cyanobacteria as a “cyanofactory” for sustainable bioplastic production [65]. Upscaled production could also save our world from petroleum plastics and benefits all fields of applications, especially due its low crystallinity and better thermal stability in certain applications.

## 4. Materials and Methods

### 4.1. Cyanobacterial Strain

The 40 cyanobacterial isolates from freshwater environments, solar salterns, and hot spring areas were obtained from the culture collection of the Applied Algal Research Laboratory, Chiang Mai University, Thailand. The cyanobacteria were maintained in a BG11 medium. The BG11 medium consisted of (g L^−1^): NaNO_3_, 1.5; K_2_HPO_4_, 0.04; MgSO_4_·7H_2_O, 0.075; CaCl_2_·2H_2_O, 0.036, Citric acid, C₆H₈O₇, 0.006; Ferric ammonium citrate, C_6_H_8_O_7_·Fe(III)·NH_3,_ 0.006; EDTA (disodium salt), C_10_H_14_N_2_Na_2_O_8_, 0.001; Na_2_CO_3_, 0.02; and 1mL of a trace element solution. The trace elements solution contained (in g L^−1^) H_3_BO_3_, 2.86; MnCl_2_·4H_2_O, 1.81; ZnSO_4_·7H_2_O, 0.222 g; NaMoO_4_·2H_2_O, 0.39; CuSO_4_·5H_2_O, 0.079; and Co(NO_3_)_2_·6H_2_O, 49.4 mg (RCI Labscan Limited, Bangkok, Thailand) at pH 7.1 [66,67]. The cyanobacteria were cultivated under continuous illumination (60 µmol photon m^−2^ s^−1^) at 25 °C for freshwater and halophilic cyanobacteria and at 40 °C for hot spring cyanobacteria [68].

### 4.2. Screening of Cyanobacteria for PHBV Production

The cyanobacteria isolates were cultured under heterotrophic conditions to induce PHBV production using a BG11 medium deprived of nitrogen and omitting citric acid (a described elsewhere [69]) but supplemented with organic carbon sources from sodium acetate, CH_3_COONa, and sodium propionate, CH_3_CH_2_COONa, at 0.4% (*w*/*v*) each (≥99.0% purity; Sigma-Aldrich, St. Louis, MO, USA). The cyanobacteria isolates were incubated for 6 days and harvested via centrifugation. The pellets were completely dried at 60 °C before being stored at 20 °C for further analysis. The cyanobacterium with the most attractive rate of PHBV accumulation and an extensive 3-hydroxyvalerate mole fraction was chosen for further studies.

### 4.3. Quantification of PHBV

PHBV was quantified by measuring the methyl esters prepared by a methanolysis reaction. Briefly, 20–40 mg of dried cyanobacterial cells was added into a mixture of 1 mL each of chloroform, CHCl_3_, and acidified methanol (30% *v*/*v* sulfuric acid, H_2_SO_4_) (RCI Labscan Limited, Bangkok, Thailand) and incubated at 100 °C for 3 h. Next, phase separation was achieved by adding 1 mL each of deionized water (RCI Labscan Limited, Bangkok, Thailand) and chloroform. One microliter of the bottom phase was directly injected into the gas chromatography (GC) column using an Agilent GC 6890N equipped with a HP-5 capillary column (30 m × 0.32 mm × 0.25 µm) (Agilent Technologies, Santa Clara, CA, USA) along with a flame ionization detector. The injector temperature was set to 250 °C, and the initial temperature was 50 °C. The analytes were held for 30 s before ramping to 100 °C at 10 °C min^−1^. Finally, the temperature was increased to 290 °C at 70 °C min^−1^. Commercial standard PHBV with a natural origin and PHV content of 12 mol% (Sigma-Aldrich, St. Louis, MO, USA) was used as a quantification reference. Benzoic acid methyl ester, C_6_H_5_COOCH_3_ (≥ 99.0% purity; Sigma-Aldrich, St. Louis, MO, USA), was used as an internal standard. All the PHBV, 3HB, and 3HV contents were calculated using the area under the peak in compared to that of the known standard concentrations, represented as the percentage of weight of the polymer to dry biomass (% *w*/*w*). The 3HB and 3HV fractions were recorded as the mol% in the whole polymer. The PHA productivity was determined by calculating the PHB/PHBV content and dry biomass.

### 4.4. Growth and Biomass Measurement

The selected cyanobacterium was cultured in a BG11 medium to determine the stationary phase. The growth of the cell concentration was measured using a Ultrospec 1100 pro spectrophotometer (GE Healthcare, Chicago, IL, USA) at an absorbance of 560 nm. The biomass dry cell weight was measured via gravimetric analysis [70]. Briefly, 5 mL of the cyanobacterium suspension was filtered using pre-weighed GF/C Whatman filter paper (Whatman, Maidstone, UK). The cells were then rinsed with deionized water and kept in a 60 °C oven until the weight was constant. The dry biomass is expressed in grams per liter.

### 4.5. First-stage Cultivation: Biomass Optimization

The concentrations of the macronutrients, including NaNO_3_, K_2_HPO_4,_ and NaHCO_3,_ affecting biomass production were optimized using first-stage cultivation with a five-level-three-factor central composite rotary design (CCRD) via the software Design-Expert 11.0.5.0^®^ (Stat-Ease Inc., Minneapolis, MN, USA). Concentration ranges of 0.10–6.00 g L^−1^ for NaNO_3_, 0.01–0.30 g L^−1^ for K_2_HPO_4_, and 0.01–0.15 g L^−1^ for NaHCO_3_ were used as the inputs of the program, with 5 levels (−α, −1, 0, +1, +α) for each factor. All suggested combinations of concentrations among all macronutrients were used for the cyanobacterial growth measurements.

### 4.6. Mathematical Analysis of the Three Factors

The relationship between the three factors was mathematically established in a quadratic equation via the response surface methodology (RSM). The data from the experiments were in accordance with the model of a full second-order polynomial equation. The three factors are presented here as X_1_, X_2_ and X_3_. The second-order polynomial equation was applied as follows:

(2) $$Y\;=\;A_{0}+A_{1}X_{1}+A_{2}X_{2}\;+A_{3}X_{3}\;+A_{12}X_{1}X_{2}+A_{13}X_{1}X_{3}+A_{23}X_{2}X_{3}+A_{11}X_{21}\;+A_{22}X_{22}+\;A_{33}X_{23}$$

where Y is the corresponding factor; and X_1_, X_2,_ and X_3_ are the actual values of the independent factors. A_0_ is a constant, while A_1_, A_2,_ and A_3_ are the linear coefficients. A_12_, A_13_, and A_23_ are the interaction coefficients; and A_11_, A_22,_ and A_33_ are the quadratic coefficients.

After completing all experimental combinations, the results were used to optimize the NaNO_3_, K_2_HPO_4,_ and NaHCO_3_ concentrations for biomass production. The dry cell weight was calculated to illustrate the dependent factor of responses (Y) in biomass production. The suitable point of each factor was determined with an accurate response “numerical optimization” analysis. A combination of the predicted concentrations with the highest biomass production was tested to verify the model.

The harvested cyanobacterial biomass from the optimized first-stage cultivation was transferred to secondary-stage cultivation to induce PHBV production. To investigate the effect of the parameters influencing PHBV production, the single factor experiment under heterotrophic conditions in a BG11 medium was supplemented with 0.4% (*w*/*v*) carbon sources including glucose, glycerol (RCI Labscan Limited, Bangkok, Thailand), sodium acetate, sodium propionate, and levulinic acid, CH_3_COCH_2_CH_2_COOH (≥99.0%; Sigma-Aldrich, St. Louis, MO, USA). Nitrogen and phosphorus limitations were also studied using a BG11 medium without NaNO_3_ and K_2_HPO_4_. The 14-day period of cultivation enabled PHBV production. Immediately after PHBV extraction (Section 2.8), GC was used to quantify the biosynthesized PHBV. All tests were performed in triplicate.

### 4.7. Extraction of PHBV

Dry cyanobacterial biomass was suspended in methanol overnight at 4 °C to eliminate pigments. Each pellet was collected and dried at 60 °C. PHBV extraction was done using the methods described previously [71]. Briefly, the cyanobacterial pellet was transferred into a screw cap tube filled with chloroform and allowed to boil with continuous shaking for 3 h. After cooling, the mixture was added into hexane to precipitate the PHBV, which was collected via centrifugation at 5000 rpm for 5 min. The crude PHBV polymer was cleaned 3 times with acetone, placed in a glass Petri dish, and stored at 4 °C for further analysis.

### 4.8. Scanning Electron Microscopy (SEM) Analysis

Surface analysis of the extracted PHBV was carried out using SEM (JEOL-JSM-5410LV, JEOL LTD., Tokyo, Japan). Prior to analysis, a sputter coater (Polaron-SC7640, Quorum Technologies, Lewes, UK) was used to apply a gold coating to the sample.

### 4.9. Mass Spectrometry Analysis

Agilent GC-MS (Agilent Technologies, Santa Clara, CA, USA) was used to qualify the PHBV. In addition to the GC protocol described previously (Section 4.3), a 2.0 min solvent delay was used, and the mass spectra were obtained under a scanning mode in the range of 40–600 *m*/*z*. Identification of the tested molecule was facilitated via the NIST11 library.

### 4.10. H-NMR and 13C-NMR Analysis

The extracted cyanobacteria polymer was suspended in deuterochloroform, CDCl_3_ (Sigma-Aldrich, St. Louis, MO, USA), at a concentration of 10 mg mL^–1^. The chemical structure was characterized using the 1H and 13C resonance frequencies. The spectra were obtained using a Bruker AVANCE 400 MHz NMR with a TOPSPIN (PS751) spectrometer at 25 °C [27]. Tetramethylsilane, Si(CH_3_)_4_ (Supelco, Sigma-Aldrich, St. Louis, MO, USA), was used as the internal shift standard.

### 4.11. Fourier-Transform Infrared (FTIR) Spectroscopy

The FTIR spectra of the extracted PHBV were recorded using a Nicolet 6700 FT-IR spectrometer (ThermoFisher Scientific, Waltham, MA, USA) equipped with a diamond crystal single bounce attenuated total reflectance (ATR) attachment. Each spectrum was obtained with a repetition of 64 scans in the range of 400–4000 cm^−1^ with a resolution of 4 cm^−1^ at room temperature.

### 4.12. Gel Permeation Chromatography (GPC)

The weight and number-average molecular weight (Mw and Mn), as well as the polydispersity index (PDI), were evaluated via GPC (Waters, Milford, MA, USA), as described [27]. The extracted PHBV was dissolved in chloroform at a concentration of 5 mg mL^−1^. Chloroform was used in the continuous phase at a flow rate of 1.0 mL min^−1^. Polystyrene was used with the mass standards.

### 4.13. Thermal Properties

The thermal properties, including the melting temperature (*Tm*), thermal crystallization temperature (*Tc*), and glass transition temperature (*Tg*), were analyzed using differential scanning calorimetry (DSC) (Mettler Toledo, Columbus, OH, USA), as described elsewhere [72]. Briefly, PHBV pellets weighing approximately 10 mg were inserted into the specimen pan. The DSC temperature ranged from −50 to 250 °C along with 3 cycles under a nitrogen atmosphere (1st cycle used ambient heating up to 250 °C; the 2nd cycle used cooling from 250 to −50 °C; and the 3rd cycle used heating from −50 to 250 °C). A heating rate of 10 °C/min. and cooling rate of −5 °C/min were applied during the DSC runs. The STAR^e^ Evaluation software was used for the thermogram analysis. The degree of crystallinity (*Xc*, %) was calculated according to the following relation [55]:

(3) $$Xc\;\left(\%\right)=\;\frac{\Delta Hm\;\times 100}{\Delta H˚m}$$

where ΔH_m_ is the melting enthalpy of the extracted PHBV, and ΔH˚_m_ is the melting enthalpy of 100% crystalline PHB, presuming 146 J g^−1^ [23]. The crystallizable fraction (CF%) was calculated with the following equation [73]:

(4) $$\mathrm{CF}\;(\%)=\;\frac{\Delta \mathrm{Hc}\times 100}{\Delta {\mathrm{Hm}}_{}}.$$

where ΔH_c_ is the crystallization enthalpy of the extracted PHBV.

The temperature at 5% weight loss (*T_d(5%)_*) and the maximum degradation temperature (*T_max_*) were evaluated via thermogravimetric analysis (TGA) using a TG-DTA-8122-Evo2G (Rigaku Corporation, Tokyo, Japan). The extracted 10 mg of PHBV was heated at a rate of 10 ˚C min^−1^ from room temperature to 500 °C [23].

## 5. Conclusions

This study found that *Cyanosarcina* sp. AARL T020, a hot spring cyanobacterium, has the greatest capability to accumulate the bioplastic polymer known as PHBV under two-stage cultivation to achieve a high rate of biomass production. Moreover, PHBV productivity indicated a greater proportion of 3HV than previously reported. The *Cyanosarcina* sp. AARL T020 presented 4.88-fold greater biomass compared to the standard trace element BG11 medium under optimized NaNO_3_, K_2_HPO_4_, and NaHCO_3_, concentrations at 4.35, 0.20, and 0.09 g L^−1^, respectively. Furthermore, PHBV production of 69.18%, comprising a 94.09 mol% 3HV fraction, is attractive for up-scaling cultivation with PHBV productivity of 81.30 mg L^−1^ day^−1^, which is reasonable for industrial production. The low crystallinity and specific thermal properties were useable in particular applications. However, other physical factor optimizations and PHBV mechanical properties must be further investigated for future advanced applications.

## Figures and Tables

**Table 1:** Differential experimental range and level of independent variables.

Independent Variables	Symbol Coded	Level of Variables				
		(−α)	−1	0	+1	(+α)
NaNO_3_ (g L^−1^)	X1	0.10	1.30	3.05	4.80	6.00
K_2_HPO_4_ (g L^−1^)	X2	0.01	0.07	0.15	0.24	0.30
NaHCO_3_ (g L^−1^)	X3	0.01	0.04	0.08	0.12	0.15

**Table 2:** ANOVA for the experimental results of the central composite design belonging to biomass production.

Source	Sum of Squares	df	Mean Square	F Value	*p*-Value Prob > F	
Model	0.25	9	0.028	7.37	0.0077	Significant
A	0.045	1	0.045	11.83	0.0108	Significant
B	0.099	1	0.099	26.24	0.0014	Significant
C	4.727 × 10^−3^	1	4.727 × 10^−3^	1.25	0.3007	
A^2^	0.014	1	0.014	3.66	0.0973	
B^2^	0.074	1	0.074	19.54	0.0031	Significant
C^2^	0.040	1	0.040	10.65	0.0138	Significant
AB	1.125 × 10^−4^	1	1.125 × 10^−4^	0.030	0.8680	
AC	1.513 × 10^−3^	1	1.513 × 10^−3^	0.40	0.5475	
BC	0.011	1	0.011	2.78	0.1396	
Residual	0.027	7	3.787 × 10^−3^			
Lack of Fit	8.307 × 10^−3^	5	1.661 × 10^−3^	0.18	0.9450	Not significant
Pure Error	0.018	2	9.100 × 10^−3^			
Cor Total	0.28	16				

R^2^ = 0.9045, adjusted R^2^ = 0.7817, predicted R^2^ = 0.6247, coefficient of variance = 7.88%.

**Table 3:** First-stage biomass optimization of *Cyanosarcina* sp. AARL T020 cultivation.

Independent Variables (g L^−1^)	Before Optimization	After Optimization	Biomass Production (g L^−1^)	
			Before optimization	After optimization
X1: NaNO_3_	1.50	4.35	0.250 ± 0.03	1.220 ± 0.07
X2: K_2_HPO_4_	0.04	0.20		
X3: NaHCO_3_	0.02	0.09		

**Table 4:** PHBV production by *Cyanosarcina* sp. AARL T020 during second-stage cultivation under heterotrophic conditions for 14 days after optimizing the culture for 14 days. The contents of the 3HB and 3HV subunits were also identified after supplementation with 0.4% (*w*/*v*) of a carbon source influencing PHBV production.

Condition /Supplementation	Polymer Type	Polymer Content (%)	3HB Fraction (mol%)	3HV Fraction (mol %)	Dry Biomass (mg L^−1^)	PHBV or PHB Productivity (mg L^−1^ day^−1^)
Control	ND	ND	ND	ND	958.00 ± 83.80 ^b^	ND
Nitrogen limitation	ND	ND	ND	ND	919.17 ± 191.44 ^b^	ND
Phosphorus limitation	ND	ND	ND	ND	970.33 ± 139.50 ^b^	ND
Glucose	PHB	2.83 ± 0.28 ^a^	100 ^a^	ND	556.00 ± 115.61 ^a^	1.11 ± 0.17 ^a^
Sodium acetate	PHB	12.34 ± 0.83 ^b^	100 ^a^	ND	1519.67 ± 38.42 ^d^	13.41 ± 1.20 ^b^
Glycerol	PHB	17.92 ± 0.79 ^c^	100 ^a^	ND	1207.67 ± 49.80 ^c^	15.43 ± 0.20 ^b^
Sodium propionate	PHBV	3.28 ± 0.71 ^a^	20.92 ± 13.32 ^c^	79.08 ±13.32 ^a^	774.67 ± 24.40 ^ab^	1.81 ± 0.39 ^a^
Levulinic acid	PHBV	69.18 ± 2.06 ^d^	5.91 ± 0.72 ^b^	94.09 ± 0.72 ^b^	1641.33 ± 128.59 ^d^	81.30 ± 8.80 ^c^

All values are the mean ± SE, *n* = 3. The values in each column are indicated by letters that are significantly (*p* < 0.05) different from each other following Duncan’s new multiple range test. Each column was analyzed separately. N.D., no detected polymer production according to GC analysis.

**Table 5:** PHBV productions in various organisms under different cultural conditions; *Tm*, melting temperature; *Tg*, glass transition temperature; *Tc*, thermal crystallization temperature; *T_d(5%)_*, the temperature at 5% weight loss; *Tmax*, the maximum degradation temperature; *ΔHm*, melting enthalpy; *Xc*, degree of crystallinity; *Mw*, weight-average molecular weight; *Mn*, weight-average molecular weight.

Organisms	Condition/Supplementation	PHBV Production				Thermal Properties						Crystallinity Index	Molecular Weight			References
		PHBV Content (%)	PHBV Productivity (mg L^−1^ day^−1^)	3HB Fraction (mol%)	3HV Fraction (mol %)	*T_m_* (°C)	*T_g_* (°C)	*T_c_* (°C)	*T_d(5%)_* (°C)	*T_max_* (°C)	ΔH_m_ (J g^−1^)	*X_c_* (%)	*M_w_* (kDa)	*M_n_* (kDa)	*M_w_/M_n_*	
Commercial PHBV ^a^	NA	NA	NA	92	8.00	167.1	24.9	115.7	NA	290	131.6	90.1	NA	NA	NA	[51]
*Oscillatoria okeni*	Nitrogen limitation, acetate	42.00	66.83	93.50	6.50	168	−3.3	53	NA	NA	65	44	54.3	32.1	1.70	[18]
*Nostoc muscorum*	Acetate and propionate	42.40	8.90	78.00	22.00	148.8	−4.3	NA	NA	NA	NA	NA	NA	NA	NA	[16]
*Aulosira fertilisima*	valerate	24.80	29.84	45.90	54.10	NA	NA	NA	NA	NA	NA	NA	NA	NA	NA	[17]
*Nostoc muscorum*	Phosphorus limitation, acetate, glucose and valerate	71.00	98.30	72.80	27.20	145	−4.7	NA	275	291	57.7	39.5	NA	NA	NA	[23]
*Nostoc microscopicum*	Nitrogen limitation, acetate	3.40	7.83	3.80	96.20	101	−9	70	NA	NA	33	22.6	49.2	26.0	1.90	[19]
*Cyanosarcina* sp. AARL T020	Levulinic acid	69.18	81.30	5.91	94.09	121	−19	67	253	284	7.07	4.84	63.9	42.0	1.52	This study

^a^ Commercial standard PHBV (Tianan Biologic, CAS Number 1039549-27-3), natural origin, PHV content 8 mol%. NA; Not Available.

## Data Availability

All data are available in the manuscript and the supplementary materials.

## Supplementary Materials

The following are available online at https://www.mdpi.com/2223-7747/10/3/503/s1, Table S1: PHBV production screening in 40 cyanobacterial strains supplemented with 0.4% (*w*/*v*) sodium acetate and 0.4% (*w*/*v*) sodium propionate under heterotrophic conditions; Table S2: Nitrogen and phosphorus ratio compared with the standard and optimized trace element-BG11 medium; Table S3: The phototrophic growth curve of *Cyanosarcina* sp. AARL T020 in a BG11 medium; Table S4: Central composite design matrix for three variables along with the predicted and experimental values of biomass production; Table S5: The raw data for before and after dry biomass production belonging to the biomass optimization (i.e., first-stage cultivation) of *Cyanosarcina* sp. AARL T020 cultivation; Table S6: The raw data of PHBV production by *Cyanosarcina* sp. T020 in second-stage cultivation under heterotrophic conditions for 14 days after optimizing the culture for 14 days. The 3HB and 3HV subunits were also identified to influence PHBV production when supplemented with 0.4% (*w*/*v*) from a carbon source.
