# Supplementary material for: Low Crystallinity of Poly(3-Hydroxybutyrate-co-3-Hydroxyvalerate) Bioproduction by Hot Spring Cyanobacterium Cyanosarcina sp. AARL T020
Source: Plants (Basel). 2021 Mar 8;10(3):503. doi: 10.3390/plants10030503 (PMC7999023; doi:10.3390/plants10030503)
Supplement: Supplementary file 1 [file plants-10-00503-s001.pdf]

Supplementary material S1.

Table S1. PHBV production screening in 40 cyanobacterial strains supplemented with 0.4% (*w/v*) sodium acetate and 0.4% (*w/v*) sodium propionate under heterotrophic conditions.

Strain number	Species name	Polymer type	Subunit content (% in dry biomass)		3HV fraction (mol %)
			3HB	3HV	
1	*Anabaena* sp. AARL C001	N.D.	N.D.	N.D.	-
2	*Arthrospira* sp. AARL BSF	PHB	45.73	N.D.	-
3	*Arthrospira* sp. AARL C002	N.D.	N.D.	N.D.	-
4	*Arthrospira* sp. AARL C003	PHBV	25.48	0.56	2.19
5	*Arthrospira* sp. AARL C004	PHB	16.09	N.D.	-
6	*Arthrospira* sp. AARL C005	PHBV	17.05	0.26	1.52
7	*Arthrospira* sp. AARL C006	N.D.	N.D.	N.D.	-
8	*Arthrospira* sp. AARL C007	PHBV	23.23	2.07	8.91
9	*Arthrospira* sp. AARL C036	PHBV	17.33	0.54	3.11
10	*Chroococccidiopsis* sp. AARL T001	PHBV	12.43	1.09	8.76
11	*Chroococccidiopsis* sp. AARL T002	PHB	10.01	N.D.	-
12	*Chroococccidiopsis* sp. AARL T003	PHBV	27.67	0.86	3.10
13	*Chroococcus* sp. AARL T004	PHBV	10.46	1.37	13.09
14	*Chroococcus* sp. AARL T005	N.D.	N.D.	N.D.	-
15	*Chroococcus* sp. AARL C023	N.D.	N.D.	N.D.	-
16	*Chroococcus* sp. AARL C032	N.D.	N.D.	N.D.	-
17	*Cyanosarcina* sp. AARL T018	PHB	9.87	N.D.	-
18	*Cyanosarcina* sp. AARL T019	PHBV	12.55	0.66	5.25
19	*Cyanosarcina* sp. AARL T020	PHBV	28.73	4.72	16.42
20	*Diclothrix* sp. AARL T021	PHBV	11.25	0.33	2.93
21	*Leptolyngbya* sp. AARL T022	PHBV	15.52	0.86	5.54
22	*Leptolyngbya* sp. AARL T023	PHBV	1.57	0.13	8.28
23	*Leptolyngbya* sp. AARL C010	N.D.	N.D.	N.D.	-
24	*Leptolyngbya* sp. AARL C011	PHBV	15.44	0.26	1.68
25	*Leptolyngbya* sp. AARL C012	N.D.	N.D.	N.D.	-
26	*Leptolyngbya* sp. AARL KC45	PHBV	11.15	0.18	1.61
27	*Leptolyngbya* sp. AARL KRABI5	N.D.	N.D.	N.D.	-
28	*Mastigocladus* sp. AARL T024	N.D.	N.D.	N.D.	-
29	*Nostoc* sp. AARL C008	N.D.	N.D.	N.D.	-
30	*Nostoc* sp. AARL C016	N.D.	N.D.	N.D.	-
31	*Nostoc* sp. AARL UP1	PHBV	37.07	0.82	2.21
32	*Nostoc* sp. AARL UP2	PHBV	26.86	0.61	2.27
33	*Nostoc* sp. AARL UP3	N.D.	N.D.	N.D.	-
34	*Phormidium* sp. AARL C009	N.D.	N.D.	N.D.	-
35	*Phormidium* sp. AARL C017	N.D.	N.D.	N.D.	-
36	*Phormidium* sp. AARL C021	N.D.	N.D.	N.D.	-
37	*Pseudanabaena* sp. AARL T025	PHBV	23.68	0.65	2.74
38	*Pseudanabaena* sp. AARL T026	PHBV	14.45	0.32	2.21
39	*Synechococcus* sp. AARL T027	PHB	10.32	N.D.	-
40	*Wolea* sp. AARL C040	N.D.	N.D.	N.D.	-
*	Commercial PHBV	PHBV	88.00	12.00	12.00

* The commercial standard PHBV-ENMAT Y1000P (Sigma-Aldrich. CAS Number 80181-31-3), natural origin PHV content 12 mol %. ***N.D.* is not detectable by gas chromatography (GC).

Table S2. Nitrogen and phosphorus ratio compared with the standard and optimized trace element-BG11 medium.

Trace element-BG11 medium	Macronutrient compositions ($g\ L^{-1}$)			Nitrogen: Phosphorus ratio
	X_1: $NaNO_3$	X_2: K_2HPO_4	X_3: $NaHCO_3$	
Before optimization	1.50	0.04	0.02	37.50
After optimization	4.35	0.20	0.09	21.75

Table S3. The phototrophic growth curve of *Cyanosarcina* sp. AARL T020 in a BG11 medium.

Replication number	day												
	0	2	4	6	8	10	12	14	16	18	20	22	24
1	0.021	0.010	0.130	0.080	0.250	1.080	1.300	1.300	1.410	1.100	0.950	1.050	0.800
2	0.023	0.030	0.137	0.120	0.263	1.157	1.420	1.557	1.593	0.983	0.943	0.893	0.780
3	0.027	0.027	0.150	0.117	0.270	1.230	1.397	1.427	1.307	1.050	0.920	0.913	0.660
Average	0.024	0.022	0.139	0.106	0.261	1.156	1.372	1.428	1.437	1.044	0.938	0.952	0.747
S.D.	0.002	0.009	0.008	0.018	0.008	0.061	0.052	0.105	0.118	0.048	0.013	0.070	0.062

Table S4. Central composite design matrix for three variables along with the predicted and experimental values of biomass production.

Run No	Independent Variables			Response (Y) biomass production ($g\ L^{-1}$)	
	$NaNO_3$ ($g\ L^{-1}$)	K_2HPO_4 ($g\ L^{-1}$)	$NaHCO_3$ ($g\ L^{-1}$)	Actual	Predicted
1	3.05	0.16	0.15	0.80	0.78
2	1.30	0.24	0.12	0.81	0.85
3	3.05	0.16	0.08	0.97	0.92
4	1.30	0.07	0.12	0.59	0.60
5	6.00	0.16	0.08	0.92	0.92
6	3.05	0.16	0.08	0.98	0.92
7	3.05	0.30	0.08	0.88	0.84
8	1.30	0.24	0.04	0.68	0.71
9	4.80	0.07	0.12	0.69	0.69
10	0.10	0.16	0.08	0.77	0.73
11	4.80	0.24	0.12	0.92	0.93
12	4.80	0.07	0.04	0.76	0.75
13	3.05	0.16	0.08	0.81	0.92
14	3.05	0.01	0.08	0.55	0.55
15	3.05	0.16	0.01	0.75	0.72
16	1.30	0.07	0.04	0.58	0.60
17	4.80	0.24	0.04	0.82	0.84

Table S5. The raw data for before and after dry biomass production belonging to the biomass optimization (i.e., first-stage cultivation) of *Cyanosarcina* sp. AARL T020 cultivation.

Independent variables ($g\ L^{-1}$)	Before optimization	After optimization	Dry biomass ($g\ L^{-1}$)		
			Replication number	Before optimization	After optimization
X1: $NaNO_3$	1.50	4.35	1	0.239	1.141
X2: K_2HPO_4	0.04	0.20	2	0.302	1.193
X3: $NaHCO_3$	0.02	0.09	3	0.209	1.327
			Average	0.250	1.220
			S.D.	0.039	0.078

Table S6. The raw data of PHBV production by *Cyanosarcina* sp. T020 in second-stage cultivation under heterotrophic conditions for 14 days after optimizing the culture for 14 days. The 3HB and 3HV subunits were also identified to influence PHBV production when supplemented with 0.4% (w/v) from a carbon source.

Condition /Supplementation	Replication number	Polymer type	Polymer content (%)	3HB fraction (mol%)	3HV fraction (mol %)	Dry biomass (mg L-1)	PHBV or PHB productivity ($mg\ L^{-1}\ day^{-1}$)
Control	1	ND	ND	ND	ND	921.00	ND
	2	ND	ND	ND	ND	879.00	ND
	3	ND	ND	ND	ND	1074.00	ND
	Average	-	-	-	-	958.00	-
	S.D.	-	-	-	-	83.80	-
Nitrogen limitation	1	ND	ND	ND	ND	731.50	ND
	2	ND	ND	ND	ND	844.00	ND
	3	ND	ND	ND	ND	1182.00	ND
	Average	-	-	-	-	919.17	-
	S.D.	-	-	-	-	191.44	-
Phosphorus limitation	1	ND	ND	ND	ND	915.00	ND
	2	ND	ND	ND	ND	834.00	ND
	3	ND	ND	ND	ND	1162.00	ND
	Average	-	-	-	-	970.33	-
	S.D.	-	-	-	-	139.50	-
Glucose	1	PHB	2.54	100.00	ND	595.00	1.08
	2	PHB	3.21	100.00	ND	399.00	0.91
	3	PHB	2.75	100.00	ND	674.00	1.32
	Average		2.83	100.00	-	556.00	1.11
	S.D.		0.28	0.00	-	115.61	0.17

Table S6. (continue): The raw data of PHBV productions by *Cyanosarcina* sp. T020 in the second -stage cultivation under heterotrophic condition for 14 days after optimized culture for 14 days. 3HB and 3HV subunits were also identify for their contents when supplemented with 0.4% (w/v) of carbon source influencing PHBV production.

Condition /Supplementation	Replication number	Polymer type	Polymer content (%)	3HB fraction (mol%)	3HV fraction (mol %)	Dry biomass (mg L^{-1})	PHBV or PHB productivity (mg L^{-1} day^{-1})
Sodium acetate	1	PHB	11.65	100.00	ND	1469.00	12.22
	2	PHB	13.50	100.00	ND	1562.00	15.06
	3	PHB	11.87	100.00	ND	1528.00	12.96
	Average		12.34	100.00	-	1519.67	13.41
	S.D.		0.83	0.00	-	38.42	1.20
Glycerol	1	PHB	18.95	100.00	ND	1154.00	15.62
	2	PHB	17.76	100.00	ND	1195.00	15.16
	3	PHB	17.04	100.00	ND	1274.00	15.51
	Average		17.92	100.00	-	1207.67	15.43
	S.D.		0.79	0.00	-	49.80	0.20
Sodium propionate	1	PHBV	2.48	25.40	74.60	799.50	1.42
	2	PHBV	3.17	2.84	97.16	741.50	1.68
	3	PHBV	4.20	34.52	65.48	783.00	2.35
	Average		3.28	20.92	79.08	774.67	1.81
	S.D.		0.71	13.32	13.32	24.40	0.39
Levulinic acid	1	PHBV	71.98	6.67	93.33	1805.50	92.83
	2	PHBV	67.08	4.93	95.07	1491.50	71.46
	3	PHBV	68.49	6.13	93.87	1627.00	79.60
	Average		69.18	5.91	94.09	1641.33	81.30
	S.D.		2.06	0.72	0.72	128.59	8.80
